# Research into the (Cost-) effectiveness of the ketogenic diet among children and adolescents with intractable epilepsy: design of a randomized controlled trial

**DOI:** 10.1186/1471-2377-11-10

**Published:** 2011-01-25

**Authors:** Reina JA de Kinderen, Danielle AJE Lambrechts, Debby Postulart, Alfons GH Kessels, Jos GM Hendriksen, Albert P Aldenkamp, Silvia MAA Evers, Marian HJM Majoie

**Affiliations:** 1 Care and Public Health Research Institute (CAPHRI), Maastricht University, Maastricht, The Netherlands; 2 Department of Research and Development, Epilepsy Centre Kempenhaeghe, Heeze, The Netherlands; 3 Department of Neurology, Epilepsy Centre Kempenhaeghe, Heeze, The Netherlands; 4 Department of Clinical Epidemiology and Medical Technology Assessment, University Hospital Maastricht, Maastricht, The Netherlands; 5 Department of Behavioral Sciences, Epilepsy Centre Kempenhaeghe, Heeze, The Netherlands; 6 Department of Neurology, Maastricht University Medical Centre, Maastricht, The Netherlands; 7 Research School of Mental Health & Neuroscience, Maastricht University, Maastricht, The Netherlands

## Abstract

**Background:**

Epilepsy is a neurological disorder, characterized by recurrent unprovoked seizures which have a high impact on the individual as well as on society as a whole. In addition to the economic burden, epilepsy imposes a substantial burden on the patients and their surroundings. Patients with uncontrolled epilepsy depend heavily on informal care and on health care professionals. About 30% of patients suffer from drug-resistant epilepsy. The ketogenic diet can be a treatment of last resort, especially for children. The beneficial effect of the ketogenic diet has been proven, but information is lacking about its cost-effectiveness. In the current study we will evaluate the (cost-) effectiveness of the ketogenic diet in children and adolescents with intractable epilepsy.

**Methods/Design:**

In a RCT we will compare the ketogenic diet with usual care. Embedded in this RCT will be a trial-based and model-based economic evaluation, looking from a societal perspective at the cost-effectiveness and cost-utility of the ketogenic diet versus usual care. Fifty children and adolescents (aged 1-18) with intractable epilepsy will be screened for eligibility before randomization into the intervention or the usual care group. The primary outcome measure is the proportion of children with a 50% or more reduction in seizure frequency. Secondary outcomes include seizure severity, side effects/complaints, neurocognitive, socio-emotional functioning, and quality of life. Costs and productivity losses will be assessed continuously by a prospective diary and a retrospective questionnaire. Measurements will take place during consults at baseline, at 6 weeks and at 4 months after the baseline period, and 3, 6, 9 and 12 months follow-up after the 4 months consult.

**Discussion:**

The proposed research project will be the first study to provide data about the cost-effectiveness of the ketogenic diet for children and adolescents with intractable epilepsy, in comparison with usual care. It is anticipated that positive results in (cost-) effectiveness of the proposed intervention will contribute to the improvement of treatment for epilepsy in children and adolescents and will lead to a smaller burden to society.

**Trial registration:**

The study has been registered with the Netherlands Trial Registry (NTR2498).

## Background

Epilepsy is a brain disorder characterized predominantly by recurrent and unpredictable interruptions of normal brain function. Seizures are transient occurrences of signs and/or symptoms due to such abnormal excessive or synchronous neuronal activities in the brain [1]. Different parts of the brain can be the site of such discharges. The clinical manifestations of seizures will therefore vary and depend on where in the brain the disturbance first starts and how far it spreads. Transient symptoms can occur, such as loss of awareness or consciousness and disturbances of movement, sensation, mood or mental function [1]. Furthermore, epilepsy is a major cost-intensive and worldwide health problem [2]. In 2000, the aggregate burden due to epilepsy was 0.5% of the total burden of diseases in the world [3]. In Europe, about 3.4 million people suffer from epilepsy [4]. The annual total costs of epilepsy in Europe are €15.5 billion [5]. In the Netherlands, direct medical costs were €221 million in 2005 [6]. In addition to the economic burden, epilepsy imposes a substantial burden on the patients themselves and their surroundings.

In the Netherlands, the point prevalence of epilepsy in children and adolescents between 0-18 years is 4.77 boys, and 4.55 girls per 1,000 persons. The yearly incidence of epilepsy in the Netherlands for children and adolescents between 0-18 years is 0.58 per 1,000 boys and 0.53 per 1,000 girls [7]. Although epilepsy is treatable with anti-epileptic drugs in the majority of cases, about 30% of patients suffer from drug-resistant epilepsy [8]. Patients with uncontrolled epilepsy heavily depend on informal care (family and friends) and on health care professionals (neurologists, social workers, psychologists etc.). Complications due to intractable epilepsy result in frequent hospitalizations and many of these patients are institutionalized. Patients with drug-resistant epilepsy can potentially benefit from a ketogenic diet. The ketogenic diet is a high-fat, low carbohydrate, normocaloric diet that mimics the metabolic state of fasting. During a prolonged fast, body energy requirements are met by lipolysis and ß-oxidation of fatty acids rather than by the breakdown of carbohydrates. The ketogenic diet maintains an anabolic nutritional state in a metabolic situation of fasting. Ketone bodies may produce an anticonvulsant effect, presumably due to changes in cerebral energy metabolism, in cell properties decreasing excitability, in neurotransmitter function, in circulating factors acting as neuromodulators and in the brain's extracellular milieu [9]. The ketogenic diet is generally used for a period of up to 3 years. Seizure control benefits are typically seen within 1-3 months of starting the diet. The international study group reports that the diet should be utilized for at least 3 1/2 months before deciding to discontinue it [10]. The diet can be discontinued earlier if seizures worsen beyond expectations or if adverse effects cannot be corrected [11]. Medications are tapered once the efficacy of the diet has been established (usually within 3-6 months of diet initiation). The diet is gradually tapered off by lowering its fat content and increasing the carbohydrate and protein portion of the diet until ketosis is eliminated. Tapering starts after 2 years of treatment, or earlier in case of intolerable side effects, or in case of ineffectiveness.

Currently the scientific and clinical attention paid to the role of the ketogenic diet is negligible. This means that a ketogenic diet is often overlooked and underutilized as a treatment option for children with intractable epilepsy. An important reason for this is that relatively few children and their parents can comply with the stringent diet. Therefore, physicians are often reluctant to initiate the diet. To offer more children the opportunity to benefit from a ketogenic diet, we suggest that the diet should be initiated and monitored under strictly controlled circumstances in order to maximize compliance.

The beneficial effect of a ketogenic diet has been studied in multiple observational studies [12-20], reviews [21-24] and in one randomized controlled trial [25]. Nevertheless, there is a lack of information about the cost-effectiveness of the ketogenic diet, and consequently health authorities have not been convinced of its usefulness.

The aims of our prospective study are to provide more evidence about the effectiveness and to investigate the cost-effectiveness of treatment with a ketogenic diet KD in comparison with usual care in children and adolescents with drug-resistant epilepsy who are not eligible for epilepsy surgery.

## Methods/Design

The design and methods of a randomized controlled trial (RCT) evaluating the (cost-) effectiveness of a ketogenic diet in comparison with usual care in children and adolescents with intractable epilepsy are described in this paper. This study has been approved by the ethics committee of the Academic Medical Centre Utrecht, the Netherlands.

### Research question

We defined the following research questions:

#### I) Effect evaluation

(1) What are the effects of the ketogenic diet in comparison with usual care with respect to changes in seizure frequency and seizure severity, side effects/complaints, neurocognitive, socio-emotional functioning, and quality of life?

#### II) Economic Evaluation

The economic evaluation will consist of a trial-based and a model-based economic evaluation study.

##### IIa) Trial-based economic evaluation

(1) From a social perspective, is the ketogenic diet, in comparison with usual care, preferable in terms of costs, effects and utilities?

(2) What is the incremental cost-effectiveness ratio (ICER) of the ketogenic diet in comparison with usual care?

##### IIb) Model-based economic evaluation

(1) In comparison with usual care, is the ketogenic diet preferable in terms of costs and utilities during the remaining life expectancy of the study population?

### Design

**Figure 1:**
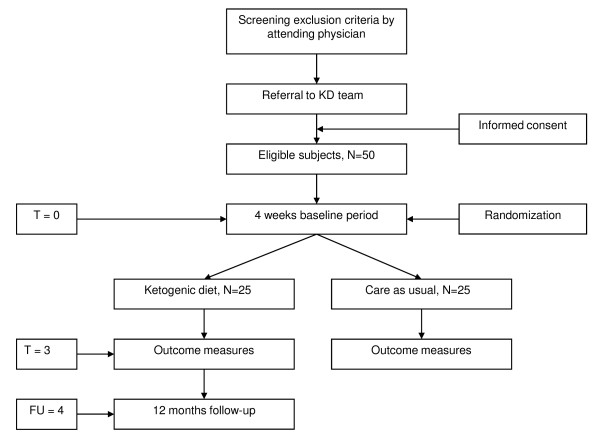
**Flowchart of the study**.

The proposed study is a prospective RCT. The subjects will be randomized to either the ketogenic diet or to usual care. The trial flow of the proposed subject enrolment and randomization procedures are shown graphically in Figure 1. Embedded in this RCT will be a short-term trial-based economic evaluation and a long-term modelling study to investigate the long-term cost-effectiveness and cost-utility of the ketogenic diet versus usual care.

### Participants

The patient population consists of children and adolescents with drug-resistant epilepsy. Patients are eligible to participate if they meet the following criteria: age between 1 and 18 years old; having uncontrolled seizures; not eligible for epilepsy surgery; no fatty acid oxidation disorders and related diseases; no diabetes and hyperinsulinism; no prolonged QT-time syndrome; no hypercholesterolemia or hypertriglyceridemia; no severe liver, kidney or pancreas diseases; no renal tubular acidosis; no severe behavioral disorder; no malnutrition; no treatment with topiramate or acetazolamide and no positive family history or other risk factors for kidney stones or acidosis.

### Recruitment

Potential participants are screened for exclusion criteria by their own attending paediatrician, neurologist or paediatric neurologist. Children who are eligible to be treated with a ketogenic diet are subsequently referred to the multidisciplinary ketogenic diet team at the epilepsy centre Kempenhaeghe in the Netherlands. Eligible children and their parents will first receive information by telephone before getting an invitation for an information visit. During the information visit eligibility is checked by the multidisciplinary team. If they are interested, parents, adolescents and/or children can find information about the study on the website http://www.ketogeenmenu.nl. Parents and potential candidates who are considering participating in the study will have at least one week to decide, before signing informed consent.

### Sample Size

A 50% reduction in seizure frequency is considered clinically relevant and is defined as success. Assuming a minimum detectable difference in success rate of 35% between the ketogenic diet group and usual care, and assuming that alpha = 5% and power = 80%, we need 22 children for each group. Taking dropouts into account, we need 50 children in total. A drop-out is defined as a child who drops out of the study before the first consultation with the neurologist; this is scheduled 6 weeks after initiating the KD or 6 weeks after randomization to usual care.

### Randomization and procedure

Randomization will occur halfway through the 4 week baseline period. We will use a software package (ALEA) to support the online patient registration and randomization which will be based on the minimization method. Patients will be stratified according to age (1-6 years, 7-12 years, 13-18 years), having a PEG/tube or not, and whether the child is living at a residential centre or attends the epilepsy centre as an outpatient while she/he lives at home. In view of the nature of the treatments, blinding of the patients and researchers is not possible.

### Intervention

#### Ketogenic diet

Patients assigned to the ketogenic diet group will be hospitalized in the tertiary epilepsy center for 1 week. During this week the ketogenic diet is introduced by a dietician. The anti-epileptic drugs the children and adolescents use at the time of inclusion in the study will be continued without changes during the study (except when medically indicated). The initial calorie prescription for the ketogenic diet is based on an average between the pre-diet intake and the recommendations for energy requirements, taking into account current and previous weight and height, recommended caloric requirements and levels of physical activity. The dietician will decide together with the parents whether the classical diet or the medium-chain triglyceride (MCT) diet is introduced. Sometimes the MCT diet is not possible and the classical ketogenic diet (3:1 or 4:1 ratio) is advised. Children with a PEG/tube feed are also treated with the ketogenic diet. The diet is then adjusted to a fluid version. It is possible to decrease the titration of anti-epileptic drugs in responders from six months after the initiation of the ketogenic diet.

#### Usual care

Patients randomized to usual care will continue to take their anti-epileptic drugs and no changes will be made to the anti-epileptic drugs treatment. Since a ketogenic diet is a treatment of last resort, the children in the usual care group will also receive a ketogenic diet after a delay of four months. The controls will be treated and monitored according to good clinical practice; however, this is not part of our proposed study.

#### Timeline of the study

**Figure 2:**
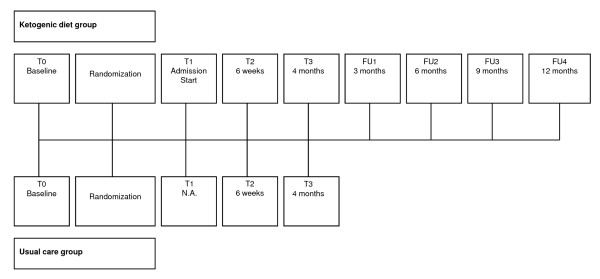
**Timeline of the study**. Note: T0 = baseline; T1 = Admission; T2 = 6 weeks after baseline period; T3 = 4 months after baseline period; FU1 = 3 months follow-up; FU2 = 6 months follow-up; FU3 = 9 months follow-up; FU12 = 12 months follow-up.

The timeline of the study is shown in Figure 2. Patients in the intervention group will visit the multidisciplinary team (nurse practitioner, dietician, paediatrician and neurologist) at time points T0 (at baseline), T1 (during admission), T2 (6 weeks after admission), T3 (4 months after admission), FU1 (3 months follow-up), FU2 (6 months follow-up), FU3 (9 months follow-up), and FU4 (12 months follow-up). At T0, T3, and FU4 a neurocognitive assessment will take place. An overview of measurements per time point is shown in Table 1. Patients in the intervention group will be asked to follow the diet during the complete study period and during follow-up, 16 months in total.

**Table 1:** Overview of measurements per time point

Measurement	T0	T1*	T2	T3	FU1*	FU2*	FU3*	FU4*
Demographic characteristics	x							

Clinical measurement	x	x	x	x	x	x	x	x

National Hospital Seizure severity scale	x	x	x	x	x	x	x	x

Side effects of Anti-Epileptic Drug	x	x	x	x	x	x	x	x

Credibility And Expectancy Questionnaire	x							

Peabody Picture Vocabulary test	x			x				x

Beery Developmental Visual-Motor Integration test	x			x				x

Fepsy computerized test	x			x				x

Kaufman Assessment Battery	x			x				x

Actigraphy	x			x				x

SEV Questionnaire	x			x				x

SDQ Questionnaire	x			x				x

POMS Questionnaire	x			x				x

The Personal Adjustment and Role Skills Scale	x			x				x

The Hague Restrictions in Childhood Epilepsy Scales	x			x				x

EuroQol	x			x				x

TAPQOL or TACQOL	x			x				x

Blood	x		x	x	x	x	x	x

Urine	x		x	x	x	x	x	x

Keton bodies measurement	x	x	x	x	x	x	x	x

ECG	x			x	x	x	x	x

T0 = baseline; T1 = Admission; T2 = 6 weeks after baseline period; T3 = 4 months after baseline period; FU1 = 3 months follow-up; FU2 = 6 months follow-up; FU3 = 9 months follow-up; FU12 = 12 months follow-up; *= intervention group only.

Patients in the usual care group will visit the multidisciplinary team (nurse practitioner, paediatrician and neurologist) at time points T0, T2, and T3 and will have a neurocognitive assessment at T0 and T3. In case of drop-out, a visit with the multidisciplinary team and a neurocognitive assessment will take place.

### I) Effect evaluation

Several instruments will be used to assess the effects of the ketogenic diet on the primary outcome (seizure frequency) and on secondary outcomes (seizure severity, side effects, neurocognitive assessment and quality of life).

#### Primary outcome

##### Seizure frequency

A seizure calendar is used to record seizures. Types of seizures are described and labelled in accordance with ILAE classification. The number and type of seizures are recorded on the dates they occur.

#### Secondary outcome

##### Seizure severity

The National Hospital Seizure severity scale (NHS3) [26] is a structured interview in which the clinician rater assigns a score to seizure severity based on interference with patient function. Information is gathered from the patient and witnesses, if available. Eight questions are asked about: tonic-clonic movements, falling, injury, incontinence, altered consciousness, postictal impairment, and disruptive automatisms.

##### Side effects

Subjective complaints are assessed using the SIDAED (Side effects of Anti-Epileptic Drug), a list of 46 items with possible anti-epileptic drug-related complaints. The items included in the SIDAED form 10 categories: general Central Nervous System (CNS), behavior (increased irritability), depressive symptoms, changes in cognitive function, motor problems and coordination, visual complaints, headache, cosmetic and dermatological complaints, gastrointestinal complaints, and sexuality and menses. For each item the parents rate the severity of the complaint on a four-point Likert scale (no problem, mild, moderate, or serious problem). In addition, the duration of the complaints is scored (for a few weeks, for months or for a half year or longer).

##### Neurocognitive assessment

*Peabody Picture Vocabulary test*

The Peabody Picture Vocabulary test (PPVT-III-NL: Dutch version) is an individually administered measure of receptive vocabulary [27]. The test can be administered beginning at the age of 2 1/2 years. The child listens to the examiner's stimulus word and must choose the picture that best describes the word from a 4-picture multiple choice array. The score on this test can be interpreted as an achievement test of the child's vocabulary that does not require a verbal response and as a screening measure of verbal ability. There is supportive data for the validity of the PPVT-III as a measure for global cognitive intelligence.

*Beery Developmental Visual-Motor Integration test*

The Beery Developmental test of Visual-Motor Integration (Beery-VMI) is a widely used assessment of psychomotor development. It measures problems with visual perception, motor coordination, and visual motor integration. The 30-item test can be administered from the age of 2 years. The Beery VMI was standardized on a national US sample of 1,737 individuals age 2 to 18 years (2010) and 1,021 adults ages 19-100 (2006), and has proven reliability and validity [28].

*Fepsy computerized test*

The Fepsy is a neurocognitive computerized test battery consisting of different subtests. For this study we use the simple auditory and visual reaction times, in which the stimulus exposure endures until a push-button (space-bar) response is given [29]. Simple reaction tests give information on alertness functions and on the speed at which the information processing system is activated. We also use the binary choice reaction task in which a combination of accuracy and speed of responses are measured. This test measures the speed of central information processing. Finally, we use the finger tapping task in which motor activation and fluency is measured. In general, these tests can be administered from the age of 6.

*Kaufman Assessment Battery*

The Kaufman Assessment Battery for Children II (K-ABC II) is used to investigate the level of information processing capacities of children beginning at the age of 2 1/2 years [30]. Two subtests will be administered: (1) Number recall, which is a measure of sequential processing and short term memory. The examiner says a series of numbers and the child repeats them. Digit span forward is considered a measure of efficiency of attention or "freedom from distractibility" and (2) conceptual thinking, which is a measure of nonverbal reasoning skills. The child looks at pictures and determines the one that does not belong.

*Actigraphy*

During the administration of the PPVT-III and the K-ABCII test, an actigraphic device (Actiwatch^® ^model AW4: Cambridge Neurotechnology Ltd., United Kingdom) will be used to assess motor activity as a measure of alertness. As some of the children in the design are not testable with the described cognitive tests we decided to use an assessment of motor activity in a standardized situation. A wrist watch will be used that translates activity into movement of the watch hand. Registration takes place in a standardized way during the first 10 minutes of the Peabody Picture Vocabulary test and the first 5 minutes of the K-ABC-II test. When patients are not eligible to perform the neurocognitive assessment, the wrist watch will be used for 15 minutes during the visit at the nurse practitioner's office while watching a DVD with songs and pictures.

*SEV Questionnaire*

The SEV questionnaire (Sociaal Emotionele Vragenlijst: Social Emotional Questionnaire) is a DSM-IV oriented questionnaire which is used to assess four domains of behavioral and social emotional dysfunction. These domains are: Attention Deficit Hyperactivity Disorders, Oppositional Defiant behavior and Conduct Disorders, Anxiety and Depression, and Autism spectrum disorders. The questionnaire consists of 72 items describing problem behavior [31].

*SDQ Questionnaire*

The Strengths and Difficulties Questionnaire (SDQ) is a brief behavioral screening questionnaire that provides balanced coverage of children and young people's behaviors, emotions, and relationships [32]. The SDQ is applicable to children beginning at the age of 4 and asks about 25 attributes that are divided into five relevant dimensions: namely conduct problems, emotional symptoms, hyperactivity, peer relationships, and pro-social behavior.

*POMS Questionnaire*

The Profile of Mood States (POMS) has been developed to identify and assess seven transient, fluctuating affective mood states: Tension-Anxiety, Depression-Dejection, Anger-Hostility, Vigour-Activity, Fatigue-Inertia, Friendliness, and Confusion-Bewilderment [33]. The questionnaire contains 65 items. All items describe an emotional state which can be rated on a 5-point scale ranging from "not at all" to "extremely".

*The Personal Adjustment and Role Skills Scale (PARS)*

The Personal Adjustment and Role Skills Scale, 3rd edition (PARS-III) was specifically developed to measure psychosocial adjustment in children with chronic physical illness [34]. This instrument is a brief parent-completed index of youth psychosocial adjustment. The instrument yields six factor-derived psychosocial subscales: namely peer relations, dependency, hostility, productivity, anxiety/depression, and withdrawal. The PARS-III is a reliable and valid index of youth psychosocial adjustment and can be used for both clinical screening and research purposes [35].

*The Hague Restrictions in Childhood Epilepsy Scales (HARCES)*

The HARCES is a questionnaire consisting of 11 items used to assess impairments in daily functioning that are related to epilepsy, e.g. how much influence does the epilepsy have on your child's freedom of functioning in the house? Items are answered on a 4-point scale ranging from "no impairment" to "very much impairment" [36].

##### Credibility And Expectancy questionnaire (CEQ)

The Credibility and Expectancy Questionnaire (CEQ) [37] is a multidimensional self-reported instrument of 6 items, rated on a 9- or 10-point Likert-scale, meant to measure the expectancy and credibility a person has about the therapy he or she will receive. Its sound psychometric properties have been proven. We will measure the credibility and expectancy for the treatment of both the children and their parents.

##### Generic quality of life

*EuroQol*

Living with uncontrolled seizures has a negative impact on the quality of life of the child and his/her parents. Therefore, we will measure generic quality of life of both the children and their parents. For this purpose we will use the EuroQol instrument for children of 12 years and older developed by the EuroQol group [38]. For younger children, we will use a version of the EuroQol developed for children (EuroQol-Youth) or proxies (Wille, 2010). In accordance with the regular EuroQol, the EuroQol-Youth is comprised of the following 5 dimensions: mobility, self-care, usual activities, pain/discomfort and anxiety/depression. Each dimension has 3 levels: no problems, some problems and severe problems, thus defining 243 (3^5^) possible health states. 'Unconscious' and 'dead' have been added to these states, resulting in a total of 245 states. Furthermore, the EQ-5 D consists of a visual analogue scale (VAS) ranging from zero (worst imaginable health state) to 100 (best imaginable health state).

*TAPQOL or TACQOL*

QoL will also be assessed using the TAPQOL (TNO-AZL Preschool Children's Quality of Life) for children aged between 1-5 years [39] and the TACQOL (TNO-AZL Children's Quality of life) for children aged between 6-16 years [40]. There are 12 dimensions in the TAPQOL: lungs, stomach, skin, sleep, appetite, aggressive behavior, positive emotions, fear, vitality, social behavior, motor functioning and communication. All dimensions are scored on a scale from 0-100. The TACQOL is comprised of 7 dimensions: bodily complaints, independence, motor functioning, cognition, social functioning, positive and negative emotions. The scores of the first 5 dimensions vary from 0-32 and the scores of the last two dimensions from 0-16.

### II) Economic evaluation

The economic evaluation will be performed from the societal perspective, which implies that all relevant costs and effects will be taken into account. The economic evaluation compares costs and effects of the ketogenic diet in comparison with usual care. A Cost-Effectiveness Analysis (CEA) and a Cost-Utility Analysis (CUA) will be performed. Outcomes of interest for the CEA and the CUA will be the reduction of seizures and improvement in the Quality Adjusted Life Years (QALYs) respectively. The QALY is a measure of disease burden, including both the quality (utilities) and the quantity of life lived. Total societal costs will be calculated based on the Dutch guidelines for cost calculations in health care. We distinguish four cost categories: intervention costs, health care sector costs, costs for the patient and family, and productivity costs. For this study, three instruments are used to measure the costs and utilities.

#### Prospective cost diary

The diary is used to identify all relevant cost aspects with respect to health care sector costs and patient and family costs [41]. Each diary covers a period of four weeks and will be filled out during the 4 week baseline period and during the 4 month study period. The intervention group will also fill out the diary during the 12 months of follow-up. Total costs will be estimated using a bottom-up approach, where information on each element of service used will be multiplied by an appropriate standardized unit cost and be summed to provide an overall total cost. For the cost valuation, standardized cost prices from the Dutch manual for cost analysis in health care research will be used [42].

#### Productivity losses

A retrospective questionnaire and the patient modules of the PROductivity and DISease Questionnaire (PRODISQ) [43] will be used to measure production losses of the patient's parents. Productivity costs will be calculated by means of the friction cost method, based on a mean added value of the Dutch working population. This method takes into account production losses confined to the period needed to replace a sick employee.

#### Utilities (EQ-5D)

Both generic quality of life, as well as utilities, will be derived by means of the EuroQol, as mentioned before. Utility values will be calculated for all health states of the EuroQol, using preferences from the UK general population value set which provides an algorithm as a series of decrements from 1, the value of full health. Values of the UK population for the scoring function of the health states described by the EQ-5D were measured with the Time Trade Off (TTO) technique (see Torrance, 1976) on a random sample of approximately 3000 members of the adult population of the UK [44]. The utility values derived from the Dolan algorithm will be used to compute Quality Adjusted Life Years (QALYs). However, to overcome the tariff's differences between countries, the Dutch tariff is also used to calculate utilities in the sensitivity analysis. Lamers [45], calculated by means of the TTO method a Dutch scoring function for the health states described by the EQ-5D, based on a Dutch population sample of 300 people.

### Analyses

#### I) Effect evaluation

Our primary (base-case) analyses will be performed according to the intention-to-treat principle, including data from all participants regardless of whether they received the intervention or not. For the analyses we will use SPSS statistical software. Respondents for whom at least 75% of the data per measurement instrument are available will be included in the analysis. Missing data on an item level will be handled using SPSS missing value regression analysis. Completely missing measurements will be handled using multiple imputation. Between-group differences in proportions (dichotomous variables) will be tested using the Chi-square test and between-group differences in means (continuous variables) will be tested using Student's t-test for independent samples. In addition, a multivariate regression analysis will be performed with the covariates of sex, age, severity of disease, duration of disease etc. The accuracy of the findings will be expressed in terms of 95% confidence intervals.

#### IIa) Trial-based economic evaluation

A baseline analysis will be performed to examine the comparability of groups at baseline for both costs and outcomes. If necessary, methods will be applied to control for differences in baseline [46]. A Kolmogorov-Smirnov test will be performed to investigate whether data are distributed normally. Despite the usual skewness in the distribution of costs, the arithmetic means will generally be considered the most appropriate measures for describing cost data [47,48]. Therefore, arithmetic means (and standard deviations) will be presented. In case the cost data are skewed, non-parametric bootstrapping will be used to test for statistical differences in costs between the intervention and usual care. The bootstrap replications will be used to calculate 95% confidence intervals around the costs (95% CI), based on the 2.5th and 97.5th percentiles. If cost data are distributed normally, t-tests will be used. The incremental cost effectiveness ratio (ICER) will be determined on the basis of the incremental costs and effects of the ketogenic diet in comparison with a waiting-list group. The cost-effectiveness ratio will be stated in terms of costs per outcome rate (decrease in seizure frequency and severity), and the cost-utility ratio will focus on the net cost per utility gained.

The robustness of the ICER will be checked by non-parametric bootstrapping. Bootstrap simulations will also be conducted in order to quantify the uncertainty around the ICER, yielding information about the joint distribution of cost and effect differences. The bootstrapped cost-effectiveness ratios will be plotted subsequently in a cost effectiveness plane. The choice of treatment depends on the maximum amount of money that society is prepared to pay for a gain in effectiveness, which is called the ceiling ratio. Therefore, the bootstrapped ICERs will also be depicted in a cost-effectiveness acceptability curve showing the probability that the ketogenic diet is cost-effective using a range of ceiling ratios. In addition, to demonstrate the robustness of our base-case findings, multi-way sensitivity analyses will be performed [49].

#### IIb) Model-based economic evaluation

The time horizon of the Cost Utility Analysis (CUA) will be extrapolated towards the remaining life expectancy of the study population. The CUA is of major importance since the impact of a ketogenic diet on seizure reduction, costs, and QoL reaches beyond the 4-month study period of the clinical study. A Markov Monte Carlo decision analytic model will be developed to calculate lifetime incremental costs and incremental QALYs of treatment with a ketogenic diet in comparison with treatment with anti-epileptic drugs only. Markov models assume that a patient is always in one of a finite number of health states. All events are represented as transitions from one state to another. A first-order Monte Carlo evaluation of a Markov model determines the prognoses of a large number of individual patients. The time horizon of the analysis is divided into cycles. Each patient begins in an initial state. During each cycle, the patient may make a transition from one state to another according to the laws of chance, as dictated by the transition probabilities. After the first patient has completed the simulation, another patient begins in the initial state and a new simulation is performed. This process is repeated a very large number of times, and each simulation generates a quality adjusted survival time and costs. Monte Carlo analysis, as opposed to a Markov cohort model without memory, offers the possibility to flag subjects in order to track their characteristics and disease histories; this is a very flexible approach to modelling variability within a population. The model will combine the results of the clinical study and data from the medical literature. In the modelling study we will also perform probabilistic sensitivity analysis to test parameter uncertainty and to construct cost-effectiveness acceptability curves. Future costs and effects will be discounted according to the Dutch guidelines for cost calculations in health care [42].

## Discussion

Our design is aimed at assessing the effectiveness and cost-effectiveness of a ketogenic diet among children and adolescents with intractable epilepsy. It is a prospective RCT comparing the ketogenic diet with usual care.

The ketogenic diet can be a treatment of last resort for patients with intractable epilepsy. This alternative therapy is used only in the minority of children who could potentially benefit from it. In order to optimize therapy for children with uncontrolled seizures, the ketogenic diet should be prescribed for more children with intractable epilepsy.

### Limitations and complications

Compliance with a ketogenic diet is difficult due to its restrictive nature. Unfortunately, non-compliance limits the intended effect and increases the costs to society, resulting in a less favorable cost-effectiveness ratio. In order to overcome this problem, the children and adolescents with uncontrolled epilepsy who are on the ketogenic diet will be monitored according to a strict standardized protocol. Therefore it is likely that the efficiency of patient care will improve. However, a formal assessment of the diet's cost-effectiveness has not yet been performed and is the focus of the present proposal.

## Conclusion

Growing up with seizures affects the child's personality and cognitive development and interferes with many aspects of everyday life, including learning at school, leisure and occupational activities. Epilepsy has been shown to have a high impact on quality of life when children have intractable seizures and additional disabilities.

This study will provide information about the cost-effectiveness of the ketogenic diet, its effects on clinical outcomes, on neurocognitive functioning and on quality of life. Our study is the first study assessing the cost-effectiveness of a ketogenic diet.

## Competing interests

The authors declare that they have no competing interests.

## Authors' contributions

RK is investigator of the cost-effectiveness part of the study and is the main author of the manuscript. DL is investigator, and grant applicator of the clinical part of the study and wrote these parts of the manuscript. DP is supervisor and grant applicator of the modelling part of the study and wrote these parts of the manuscript. AK is a local statistician and arranged the randomization procedure. JH is coordinator of the neurocognitive research. AA is professor of neuropsychology and supervisor. SE is supervisor and grant applicator of the cost-effectiveness analysis part of the study. MM is supervisor, grant applicator and senior advisor. All authors read, edited and approved the final manuscript.

## Pre-publication history

The pre-publication history for this paper can be accessed here:

http://www.biomedcentral.com/1471-2377/11/10/prepub
